# Exome data clouds the pathogenicity of genetic variants in Pulmonary Arterial Hypertension

**DOI:** 10.1002/mgg3.452

**Published:** 2018-08-06

**Authors:** Yeganeh Abbasi, Javad Jabbari, Reza Jabbari, Charlotte Glinge, Seyed Bahador Izadyar, Edda Spiekerkoetter, Roham T. Zamanian, Jørn Carlsen, Jacob Tfelt‐Hansen

**Affiliations:** ^1^ Heart Centre Department of Cardiology Copenhagen University Hospital, Rigshospitalet Copenhagen Denmark; ^2^ Department of Cardiology Section for Pulmonary Hypertension and Right Heart Failure Copenhagen University Hospital, Rigshospitalet Copenhagen Denmark; ^3^ LEO Pharma A/S Ballerup Denmark; ^4^ Division of Pulmonary and Critical Care Stanford University School of Medicine California; ^5^ Department of Forensic Medicine Faculty of Medical Sciences University of Copenhagen Denmark

**Keywords:** ESP, exome sequencing project, HGMD, PAH‐associated gene, pulmonary arterial hypertension

## Abstract

**Background:**

We aimed to provide a set of previously reported PAH‐associated missense and nonsense variants, and evaluate the pathogenicity of those variants.

**Methods:**

The Human Gene Mutation Database, PubMed, and Google Scholar were searched for previously reported PAH‐associated genes and variants. Thereafter, both exome sequencing project and exome aggregation consortium as background population searched for previously reported PAH‐associated missense and nonsense variants. The pathogenicity of previously reported PAH‐associated missense variants evaluated by using four *in silico* prediction tools.

**Results:**

In total, 14 PAH‐associated genes and 180 missense and nonsense variants were gathered. The *BMPR2*, the most frequent reported gene, encompasses 135 of 180 missense and nonsense variants. The exome sequencing project comprised 9, and the exome aggregation consortium counted 25 of 180 PAH‐associated missense and nonsense variants. The *TOPBP1* and *ENG* genes are unlikely to be the monogenic cause of PAH pathogenesis based on allele frequency in background population and prediction analysis.

**Conclusion:**

This is the first evaluation of previously reported PAH‐associated missense and nonsense variants. The *BMPR2* identified as the major gene out of 14 PAH‐associated genes. Based on findings, the *ENG* and *TOPBP1* gene are not likely to be the monogenic cause of PAH.

## 1 INTRODUCTION

Pulmonary arterial hypertension (PAH) is a rare, progressive, and deathly disease (Hoeper et al., 2014; Machado, 2012; Simonneau et al., 2013). Despite advances in treatment of the PAH, the mortality rate is still high (Ling et al., 2012). The incidence of PAH estimated in a wide range from 2.4 to 25 cases per million per year (Gaine & Rubin, 1998; Humbert et al., 2006; Ling et al., 2012).

PAH is subclassified into four groups: idiopathic PAH, heritable PAH, drug, and toxin induced, and lastly associated with certain other diseases (e.g. connective tissue disease, HIV infection, portal hypertension, congenital heart diseases, and schistosomiasis) (Simonneau et al., 2013).

The pathogenesis of PAH is complex, and, albeit the extensive effort, is still not completely understood (Badesch et al., 2009; Hoeper et al., 2013; Simonneau et al., 2013) Genetic studies have identified association of several genetic loci to PAH (Austin & Loyd, 2014; Rabinovitch, 2012; Simonneau et al., 2013). The bone morphogenetic protein receptor type II (*BMPR2*) plays a major role in the etiology of idiopathic and heritable PAH (Ma & Chung, 2014; Simonneau et al., 2013). Other reported PAH‐associated genes includes activin A receptor like type 1 (*ACVRL1)*, endoglin (*ENG*), *SMAD* family *1,4,* 9, caveolin 1 (*CAV1*), bone morphogenetic protein receptor type 1b (*BMPR1B)*, potassium voltage‐gated channel subfamily a member 5 (*KCNA5)*, potassium channel subfamily K member 3 (*KCNK3)*, T‐box transcription factor 4 (*TBX4)*, DNA topoisomerase 2‐binding protein 1 (*TOPBP1)*, growth differentiation factor 2 (*GDF2)* and eukaryotic translation initiation factor 2‐alpha kinase 4 (*EIF2AK4)* at a lower frequency (Machado, 2012; Soubrier et al., 2013). The genes that were classified as PAH‐associated genes in the guideline were *BMPR2*,* ALK‐1*,* ENG*,* SMAD9*,* CAV1*, and *KCNK3* (Simonneau et al., 2013). Genetic testing has become an important tool in the clinical evaluation of PAH patients, especially for patients with a positive family history of PAH. Therefore, it is important to determine whether PAH associated genes and variants are truly disease causing.

We aimed to provide an encyclopedia by gathering all previously published PAH‐associated genes and variants, and further evaluate the pathogenicity of each variant by performing comprehensive *in silico* prediction analysis, together with investigating the frequency of each PAH‐associated variant in two large online exome databases.

## 2 MATERIALS AND METHODS

The Human Gene Mutation Database (HGMD), PubMed, and Google Scholar were searched for previously published PAH‐associated genes and variants until October 2016. The following queries were used: ((ʻpulmonary arterial hypertension ʼ (MeSH)) or (pulmonary arterial hypertension)), ((ʻgeneticsʼ (MeSH)) or (genetic)), ((ʻmutationʼ (MeSH)) or (mutation)) and ((ʻvariantsʼ (MeSH)) or (variants)). We revisited all identified genetic variants searching for published data on functional and familial cosegregation studies. In order to have a solid baseline, familial cosegregation was defined as at least two genotype positive family members having the same phenotype. The Hugo Genome Organisation and Gene Nomenclature Committee was used for standard nomenclature of human genes (HGNC database of human gene names | HUGO Gene Nomenclature Committee). The publicly available Ensembl genome database was used to find the location of variants in the genome and determine the amino acid changes in the protein coding regions of genes (Ensembl Genome Browser 85).

### 2.1 Exome sequencing project

In the Exome Sequencing Project (ESP), next‐generation sequencing of all protein coding regions in 6,503 individuals of African American (*n* = 2,203) and European American (*n* = 4,300) from different population studies have been carried out (Exome Variant Server). Clinical data were not available. The ESP was searched for previously published PAH‐associated variants. The ESP lacks the data regarding variants positioned in promotors, introns, and untranslated regions, therefore variants found in these regions were not included in present study.

All previously identified PAH‐associated variants in our investigation were subdivided into two groups; those that were identified in the ESP (ESP‐ positive) and those that were not identified in the ESP (ESP‐negative).

### 2.2 The exome aggregation consortium

In the exome aggregation consortium (ExAC) comprehend exome sequencing data from 60,706 unrelated individuals (ExAC Browser). African/African American (*n* = 5,203), Latino (*n* = 5,789), East Asian (*n* = 4,327), Finnish (*n* = 3,307), Non‐Finnish European (*n* = 33,370), South Asian (*n* = 8,256), and other (*n* = 454) nationalities are presented in the ExAC database (ExAC Browser). Like the ESP, this browser only encompasses human genome data that encodes proteins as part of several various exome‐studies on populations with specific diseases (ExAC Browser). The PAH‐associated variants were subdivided into those that were identified in the ExAC (ExAC‐positive) and those that were not identified in the ExAC (ExAC‐negative).

The ESP and ExAC databases are considered as back ground population in this study.

### 2.3 *In silico* prediction analysis

The functional effects of all missense variants were assessed by using the four prediction tools including conservation across species, Grantham Score, PolyPhen‐2 (Polymorphism Phenotyping v2), and SIFT (Sorting Intolerant from Tolerant, v5.1.1). Data for conservation across species were obtained from Ensembl, and classified as occurring at a position with no substitutions (conserved/pathogenic) or ≥1 substitutions (not conserved/benign). Grantham physicochemical values were calculated using the Grantham amino acid difference matrix. We defined a value above 100 as radical (pathogenic), and value under 100 as conservative (benign). Using PolyPhen‐2, each variant were labeled “probably damaging”, “possibly damaging”, or “benign”. Variants labeled “probably damaging” and “possibly damaging” considered “damaging” (pathogenic) in our analysis. Finally, SIFT prediction classified variants as “tolerant” (benign) or “damaging” (pathologic). In a final analysis using all prediction tools, a variant was considered pathogenic if ≥3 *in silico* prediction tools determined the variant to be pathogenic, as previously described (Giudicessi et al., 2012). Variants that predicted pathogenic by only 1 or 2 tools was considered to be variants of uncertain significance (VUS).

## 3 RESULTS

To date, 14 genes and 180 missense/nonsense variants have been identified as PAH‐associated genes and variants (Table 1).

**Table 1:** Overview of PAH‐associated genes and variants

Gene	Missense variants	Nonsense variants
*BMPR2*	86	49
*ACVRL1*	16	1
*BMPR1B*	2	–
*CAV1*	1	–
*KCNA5*	4	–
*KCNK3*	6	–
*SMAD1*	1	–
*SMAD4*	1	–
*SMAD9*	1	2
*TBX4*	3	–
*TOPBP1*	3	–
*ENG*	2	–
*GDF2*	–	1
*EIF2AK4*	–	1
14 genes	126 missense	54 nonsense

The *BMPR2* was the most frequent reported gene and encompasses 135 (missense=86 and nonsense=49) of the 180 identified missense/nonsense PAH‐associated variants (Figure). The other previously identified PAH‐associated genes: *ACVRL1*,* BMPR1B*,* CAV1*,* KCNA5*,* KCNK3*,* SMAD1*,* SMAD4*,* SMAD9*,* TBX4*,* TOPBP1*,* ENG*,* GDF2,* and *EIF2AK4* included 45 (missense=40 and nonsense=5) of the 180 missense/nonsense PAH‐associated variants (Table 1).

We performed a prediction analysis of only the missense PAH‐associated variants (*n* = 126), because the nonsense variants are classified to be damaging by nature of the uncompleted translation. By doing so, 76 of 126 missense PAH‐associated variants were predicted as pathogenic (Table 2). Accordingly, 52 of 86 PAH‐associated missense variants in *BMPR2* gene were predicted pathogenic. Prediction analyses for all PAH‐associated genes are available in the Table 2.

**Table 2:** Prediction analysis of missense variants with ≥ 3 *in silico* prediction tools agreement

Gene	Damaging	VUS	Benign
*BMPR2*	52	24	10
*ACVRL1 (ALK1)*	13	2	1
*BMPR1B (ALK6)*	0	2	0
*CAV1*	0	0	1
*KCNA5*	2	2	0
*KCNK3*	5	1	0
*SMAD1*	0	1	0
*SMAD4*	0	1	0
*SMAD9*	1	0	0
*TBX4*	2	0	1
*TOPBP1*	1	1	1
*ENG*	0	2	0
	76	36	14

By investigating the frequency of the PAH‐associated variants in the two large population databases, we found that the ESP comprised 9 of 180 variants (Table 3), while the ExAC in counted 25 PAH‐associated missens/nonsense variants (Tables 3 and 4).

**Table 3:** Exome Sequencing Project‐positive PAH‐associated variants

Gene	Variant	dbSNP ID	All allele (EA + AA)	MAF (%) (EA/AA/All)	All genotype (EA + AA)	Grantham score	PolyPhen	SIFT	Conservation	Agreement of ≥3 tools	References
*BMPR2*	p.R266T	rs374694591	C = 1/G = 13005 (0.00007)	0.0116/0.0/0.0077	CC = 0/CG = 1/GG = 6502 (0.01%)	71	Damaging	Not tolerated	Conserved	D	Machado et al. (2006)
	p.N903S	rs373725296	G = 1/A = 13005 (0.00007)	0.0116/0.0/0.0077	GG = 0/GA = 1/AA = 6502	46	Damaging	Tolerated	Conserved	VUS	Thomas et al. (2009)
*CAV1*	p.V155I	rs150368249	A = 6/G = 13000 (0.00046)	0.0349/0.0681/0.0461	AA = 0/AG = 6/GG = 6497	29	Benign	Tolerated	Not conserved	B	Austin et al. (2012)
*KCNA5*	p.E211D	rs35853292	C = 15/G = 12991 (0.00115)	0.1512/0.0454/0.1153	CC = 0/CG = 15/GG = 6488	45	Benign	Tolerated	Conserved	VUS	Remillard et al. (2007)
*TOPBP1*	p.S817L	rs17301766	A = 1921/G = 10445 **(0.18391)**	19.4923/7.2995/15.5345	AA = 182/AG = 1557/GG = 4444	145	Benign	Tolerated	Not conserved	VUS	de Jesus Perez et al. (2014)
	p.N1042S	rs10935070	C = 2869/T = 8855 **(0.32399)**	32.7297/5.8791/24.4712	CC = 438/CT = 1993/TT = 3431	46	Benign	Tolerated	Not conserved	B	
	p.R309C	rs55633281	A = 779/G = 10999 **(0.07082)**	7.629/4.343/6.614	AA = 22/AG = 735/GG=5132	180	Damaging	Tolerated	Conserved	D	
*ENG*	p.G214S	rs150932144	T = 3/c = 12821 (0.00023)	0.0118/0.046/0.0234	TT = 0/TC = 3/CC = 6409	56	Damaging	Tolerated	Not conserved	VUS	Pfarr et al. (2013)
	p.G545S	rs142896669	T = 8/C = 12998 (0.00061)	0.0814/0.0227/0.0615	TT = 0/TC = 8/CC = 6495	56	Damaging	Tolerated	Conserved	VUS	

Note

B: benign; D: damaging; VUS: variants with uncertain significance.

**Table 4:** Exome aggregation consortium‐positive varian

Gene	Variant	rs ID	Allele frequency	Grantham score	PolyPhen	SIFT	Conservation	Agreement ≥3 tools	References
*BMPR2*	p.Q92H	rs140683387	0.000107	24	Benign	Tolerated	Not conserved	B	Kabata et al. (2013)
	p.W508*	X	0.000008	–	–	–	–	–	Pfarr et al. (20110
	p.R591*	X	0.000016	–	–	–	–	–	Sztrymf et al. (2008)
	p.T766A	X	0.000008	58	Benign	Tolerated	Not conserved	B	Liu et al. (2012)
	p.R873Q	rs201781338	0.000115	43	Damaging	Tolerated	Conserved	VUS	Sztrymf et al. (2008)
	p.R266T	rs374694591	0.000041	71	Damaging	Not tolerated	Conserved	D	Machado et al. (2006)
	p.R303H	rs200948870	0.000033	29	Damaging	Tolerated	Conserved	VUS	Machado et al. (2006)
	p.V563M	X	0.000008	21	Damaging	Tolerated	Conserved	VUS	Machado et al. (2006)
	p.R899P	rs137852752	0.000008	103	Damaging	Tolerated	Not conserved	VUS	Vattulainen et al. (2015)
	p.A24E	X	0.000008	107	Benign	Tolerated	Not conserved	VUS	Machado et al. (2009)
	p.N903S	rs373725296	0.000016	46	Damaging	Tolerated	Conserved	VUS	Thomas et al. (2009)
	p.E427D	X	0.000008	45	Benign	Tolerated	Conserved	VUS	van der Bruggen et al. (2016)
*CAV1*	p.V155I	rs150368249	0.000585	29	Benign	Tolerated	Not conserved	B	Austin et al., 2012;
*KCNA5*	p.E211D	rs35853292	0.000833	45	Benign	Tolerated	Conserved	VUS	Remillard et al. (2007)
	p.G182R	X	0.000141	125	Damaging	Not tolerated	Conserved	D	
*SMAD4*	p.N13S	rs281875323	0.000024	46	Damaging	Tolerated	Not conserved	VUS	Nasim et al. (2011)
*SMAD9*	p.K43E	X	0.000107	56	Damaging	Not tolerated	Conserved	D	Nasim et al. (2011)
	p.R294*	X	0.000008	–	–	–	–	–	Drake et al. (2011)
*TBX4*	p.A35V	rs148424252	0.007833	64	Benign	Tolerated	Not conserved	B	Kerstjens‐Frederikse et al., 2013; (p4)
	p.Y382S	X	0.000041	144	Damaging	Not tolerated	Conserved	D	
*TOPBP1*	p.S817L	rs17301766	**0.1411**	145	Benign	Tolerated	Not conserved	VUS	de Jesus Perez et al. (2014)
	p.R309C	rs55633281	**0.0533**	180	Damaging	Tolerated	Conserved	D	
	p.N1042S	rs10935070	**0.2898**	46	Benign	Tolerated	Not conserved	B	
*ENG*	p.G214S	rs150932144	0.000155	56	Damaging	Tolerated	Not conserved	VUS	Pfarr et al. (2013)
	p.G545S	rs142896669	0.000520	56	Damaging	Tolerated	Conserved	VUS	

Note

B: benign; D: damaging; VUS: variants with uncertain significance.

In the most frequent reported PAH‐associated gene, *BMPR2*, we found 2 ESP‐positive and 12 ExAC‐positive variants of 135 variants (Tables 3 and 4).

In the literature, functional studies had been performed in 29 of 180 PAH‐associated variants (Table 5), assessing the functional properties of the resulted protein using in vivo and/or in vitro studies. All functional studies showed that mutated proteins, except 2 variants (p.Ser160Asn (rs149589961) and p.Phe392Leu) in *BMPR1B*, displayed a loss of function phenotype (Table 5).

**Table 5:** Functional studies

Gene	Amino acid substitution	Type of cell/Animal	Result	References
*ACVRL1 (ALK1)*	R484W	NIH‐3T3 fibroblasts and COS‐7 cells	Loss of function	Ricard et al. (2010)
	R484Q	NIH‐3T3 fibroblasts and COS‐7 cells	Loss of function	
	L381P	NIH‐3T3 fibroblasts and COS‐7 cells	Loss of Function	
*BMPR1B (ALK6)*	S160N	COS1 cells (in vitro)	Gain of function	Chida et al. (2012)
	F392L	COS1 Cells (in vitro)	Gain of function	
*BMPR2*	W16*	PASMCs and microvascular endothelial cells	Loss of Function	Dewachter et al. (2009)
	R491W	PASMCs and microvascular endothelial cells	Loss of Function	
	Q495*	PASMCs and microvascular endothelial cells	Loss of Function	
	S301P	PASMCs and microvascular endothelial cells	Loss of Function	
	E195*	PASMCs and microvascular endothelial cells	Loss of Function	
	S107*	PASMCs and microvascular endothelial cells	Loss of Function	
	R321*	Blood outgrowth endothelial cells (BOECs) Pulmonary artery endothelial (PAEC) & Pulmonary artery smooth muscle cells (PASMC)	Loss of Function	Drake et al. (2011), Dunmore et al. (2013)
	W9*	PASMCs	Loss of Function	Thomas et al. (2009)
	C347R	PASMCs	Loss of Function	
	C347Y	PASMCs	Loss of Function	
	N903S	PASMCs	Loss of Function	
	R899*	A mouse model (In vivo)	Loss of Function	Long et al. (2015)
*KCNA5*	E211D	COS‐1(mammalian) cells & HEK‐293*(human) cells	Loss of Function	Burg et al. (2010)
	G182R	COS‐1(mammalian) cells & HEK‐293*(human) cells	Loss of Function	
*KCNK3*	T8K	COS‐7cells	Loss of Function	Ma et al. (2013)
	G97R	COS‐7cells	Loss of Function	
	E182K	COS‐7cells	Loss of Function	
	Y192C	COS‐7cells	Loss of Function	
	G203D	COS‐7cells	Loss of Function	
	V221L	COS‐7cells	Loss of Function	
*SMAD1*	V3A	PASMCs	Loss of Function	Nasim et al. (2011)
*SMAD4*	N13S	PASMCs	Loss of Function	Nasim et al. (2011)
*SMAD9*	K43E	PASMCs	Loss of Function	Nasim et al. (2011), Suo et al. (2013)
	C202*	COS1 Cells	Loss of Function	Shintani et al. (2009)

## 4 DISCUSSION

In this novel study, we provide the clinicians the first comprehensive evaluation tool for genetic diagnostic of PAH, by evaluating the allele frequency of previously reported PAH‐associated variants in the two large background population databases (ESP and ExAC), and also by adding *in silico* prediction analysis using an established conservative method (Abbasi et al., 2016; Jabbari et al., 2013; Risgaard et al., 2013). Surprisingly, in the literature we identified very limited data on familial cosegregation, thus, unfortunately, the familial cosegregation in our evaluation was very limited. This, however, goes hand in hand and support our findings that the identified ESP‐ and ExAC‐positive variants may not be the monogenic cause of the PAH. The pathogenic PAH‐associated variants in *BMPR2* gene have reduced penetrance and gender dependant (Austin, Loyd, & Phillips, 1993). Therefore, ESP and ExAC databases most likely include unaffected heterozygotes parents. The penetrance information for pathogenic PAH‐associated variants in *ACVRL1*,* KCNK3*,* CAV1*,* SMAD9,* and *BMPR1B* genes is unknown (Austin et al., 1993).

Our investigation supports that the *BMPR2* gene is of major importance in the development of the heritable and idiopathic PAH (Simonneau et al., 2013; Soubrier et al., 2013). According to our findings, *BMPR2* included 75% (135 of 180) of the previously reported missense/nonsense PAH‐associated variants. Familial cosegregation was only identified for three variants (p.W13*, p.E386V and p.K512T) in *BMPR2* gene (Fu et al., 2008; Hamid et al., 2010; Machado et al., 2006). Prediction analysis of *BMPR2* missense variants (*n* = 86), using agreement of ≥ 3 of 4 *in silico* prediction tools indicated that only 60.4% variants (*n* = 52) were predicted pathogenic.

The annual incidence of PAH is estimated from 2.4 to 25 cases per million per year in the general population (Gaine & Rubin, 1998; Humbert et al., 2006; Ling et al., 2012). In total, 12 variants in *BMPR2* were identified in the ESP and ExAC databases (Tables 3 and 4). This means ~9% of previously identified PAH‐associated variants in *BMPR2* were found in the background population. According to the incidence of PAH in background population, this is an expected frequency of PAH‐associated variants in *BMPR2* in the background population. The 12 identified functional studies on variants in *BMPR2* gene revealed that all mutated proteins had a loss of function phenotype (Table 5).Taken together, these findings point to a pivotal role of *BMPR2* in pathogenesis of PAH.

In contrast, we found all the three PAH‐associated missense variants (p.S817L [rs17301766], p.N1042S [rs10935070] and p.R309C [rs55633281]) in *TOPBP1* gene in the ESP and ExAC databases (de Jesus Perez et al., 2014). The allele frequency of p.S817L (in ESP = 0.1839 and in ExAC = 0.1411), p.N1042S (in ESP = 0.3239 and in ExAC = 0.2898), and p.R309C (in ESP = 0.0708 and in ExAC = 0.0533) in ESP and ExAC is very high (Tables 3 and 4; de Jesus Perez et al., 2014). Our prediction analysis showed that only p.R309C was predicted pathogenic. Since the PAH‐associated variants in the *TOPBP1* have high allele frequency in the background population (*n* = 3 variants) is not likely to be the monogenic cause of PAH. No functional studies have been reported on these variants and these are indeed needed to clarify the effect of variants in *TOPBP1* as a modifier gene in the pathogenesis of PAH. Furthermore, no familial cosegregation are reported in order to support *TOPBP1* monogenic cause of PAH (de Jesus Perez et al., 2014).

In 2013, the 5th World Symposium on Pulmonary Hypertension established the *ENG* gene to be a PAH‐associated gene, since two missense PAH‐associated variants (p.G214S [rs150932144] and p.G545S [rs142896669]) were reported (Simonneau et al., 2013). In our analysis, these two variants were predicted VUS (Table 2), questioning the pathogenicity of these variants in the PAH‐etiology. Furthermore, both the p.G214S and p.G545S were present in the ESP and ExAC databases (Tables 3 and 4). The allele frequency of p.G214S in the ESP was 0.0002 and 0.0001 in the ExAC database. The p.G545S variant found in the ESP with allele frequency 0.0006 and 0.0005 in the ExAC browser (Tables 3 and 4). Although *ENG* gene known as a PAH‐associated gene in the development of PAH, our data and analysis do not support that *ENG* variants are likely to be a monogenic or one of the major causes in the pathogenesis of PAH. It is important to perform a comprehensive functional study to determine the exact effect of the reported amino acid changes in the *ENG* and the effect of p.G214S and p.G545S in expression level of the protein.

In the literature we found five PAH‐associated variants in *SMAD* genes: *SMAD1* (p.V3A), *SMAD4* (p.N13S) and *SMAD9* (p.K43E, p.C202* and p.R294*). None of the five were found in the ESP, but we found three variants (p.N13S, p.K43E and p.R294*) in the ExAC (Table 4). Using pulmonary artery smooth muscle cells (PASMCs), Nasim M.T. et al. demonstrated that the p.V3A in *SMAD1* gene, p.N13S in *SMAD4* gene, and p.K43E in *SMAD9* gene resulted in reduced signaling activity in vitro of amino acid substitutions (Nasim et al., 2011). Another functional study analyzed the function of p.C202* in *SMAD9* (aliases: *SMAD8*) by using COS1 cells (a fibroblast‐like cell). This study revealed that the mutated protein was not able to have interaction with *SMAD4* gene (Tables 2 and 5; Shintani, Yagi, Nakayama, Saji, & Matsuoka, 2009). Although the p.V3A and p.N13S were predicted as VUS, the results of functional studies (loss of function) support the effect of these variants in the pathogenesis of PAH.

In the *ACVRL1* gene 16 missenses and one nonsense variants were reported (Table 1). Thirteen variants (81.25%) were predicted as pathogenic (Table 2). None of these variants were identified in ESP or ExAC databases. One in vitro functional study used NIH 3T3 fibroblasts and COS‐7 cells analyzing the protein expression of three PAH‐associated variants (p.L381P, p.R484Q and p.R484W) (Ricard et al., 2010). The study reported that p.R484Q and p.R484W were inactive in the transactivation step (Ricard et al., 2010). The mutated protein of p.L381P did not respond to the bone morphogenetic protein 9 (BMP9) stimulation (loss of function) (Ricard et al., 2010). These findings support the hypothesis of the role of mutated proteins in *ACVRL1* in pathogenesis of PAH, despite the lack of data of familial cosegregation.

The two PAH‐associated variants in *BMPR1B* gene were investigated in a functional study by using COS1 cells (Chida et al., 2012). They showed that amino acid changes in p.F392L and p.S160N increased the activation of proteins above wild‐type (gain of function) (Chida et al., 2012). The p.S160N and p.F392L identified are unlikely to be an important cause of development of PAH based on results of the functional study. Furthermore, the p.S160N and p.F392L were predicted VUS, which supports the result of the functional study (Table 2; Chida et al., 2012).

To describe the function of all six variants in *KCNK3*, Lijiang Ma et al. performed a functional analysis by using COS‐7 cells (Ma et al., 2013). The mutated proteins showed the loss of ion‐channel function (Ma et al., 2013). Supporting these results, our *in silico* prediction analysis predicted that all PAH‐associated variants in *KCNK3* except p.V221L were pathogenic (Table 2).

Burg ED et al. analyzed the mutated protein of p.E211D and p.G182R in *KCNA5* gene (Burg, Platoshyn, Tsigelny, Lozano‐Ruiz, & Rana, 2010). In an in vitro study, they compared the function of mutated proteins with wild type using human embryonic kidney cells (HEK‐293) and COS‐1 (Burg et al., 2010). They found that mutated proteins accelerated the inactivity of the voltage‐gated K^+^ (K(V)) channels, which have an important role in regulating PASMCs (Burg et al., 2010). These findings support the role of p.E211D and p.G182R in *KCNA5* gene as uncommon cause of the etiology of PAH, although these two variants predicted as VUS (Tables 2, 3 and 4).

Song et al. (2016) identified the p.Y311* as a heterozygote mutation in *EIF2AK4* gene in an heritable or idiopathic PAH patient. The p.Y311*/*EIF2AK4* was not present in the ESP and ExAC. A functional characterization of p.Y311* by a protein‐expression study and cosegregation analysis in a pedigree will support the role of p.Y311*/*EIF2AK4* in pathogenesis of PAH.

## 5 CONCLUSION

To our knowledge, this is the first evaluation of previously reported rare PAH‐associated genes and variants. In the literature, we found 14 genes and 180 missense/nonsense variants. *BMPR2* were identified to be the most important and common reported cause of PAH.

By using prediction analysis and the allele frequency of PAH‐associated variants in *TOPBP1* and *ENG* genes in the background population, suggests that these variants are unlikely to be the monogenic cause of the PAH pathogenesis. Further functional studies are required to clarify the function of mutated proteins.

## CONFLICT OF INTEREST

Javad Jabbari is employed at LEO Pharma A/S. There are no financial interests to report. This study was supported by research grants from the Research Foundation at the Heart Center, Rigshospitalet, Copenhagen, Denmark and the Novo Nordisk Foundation to Pr. Tfelt‐Hansen.
