# Supplementary material for: Exome data clouds the pathogenicity of genetic variants in Pulmonary Arterial Hypertension
Source: Mol Genet Genomic Med. 2018 Aug 6;6(5):835–44. doi: 10.1002/mgg3.452 (PMC6160702; doi:10.1002/mgg3.452)
Supplement: Supplementary file 1 [file MGG3-6-835-s001.docx]

**Table S1: PAH-associated variants in ExAC Browser:**

| **Gene** | **variant** | **rsID** | **Origin** | **References** |
| --- | --- | --- | --- | --- |
| ***BMPR2*** | p.Q92H | [rs140683387](https://www.ncbi.nlm.nih.gov/variation/tools/1000genomes/?chr=2&from=203332270&to=203332270&gts=rs140683387&mk=203332270:203332270\|rs140683387) | Germline | ^2,3^ |
|  | p.W508* | rs780722371 | Germline | ^3,4^ |
|  | p.R591* | rs777458559 | Germline | ^3,5^ |
|  | p.T766A | [rs758990535](https://www.ncbi.nlm.nih.gov/variation/tools/1000genomes/?chr=2&from=203420684&to=203420684&gts=rs758990535&mk=203420684:203420684\|rs758990535) | Germline | ^3,6^ |
|  | p.R873Q | rs201781338 | Germline | ^3,5,7^ |
|  | p.R266T | [rs374694591](http://www.ncbi.nlm.nih.gov/projects/SNP/snp_ref.cgi?rs=rs374694591) | Germline | ^3,8,9^ |
|  | p.R303H | [rs200948870](https://www.ncbi.nlm.nih.gov/variation/tools/1000genomes/?chr=2&from=203384865&to=203384865&gts=rs200948870&mk=203384865:203384865\|rs200948870) | Germline | ^3,8^ |
|  | p.V563M | [rs557172581](http://www.ncbi.nlm.nih.gov/projects/SNP/snp_ref.cgi?rs=rs557172581) | Germline | ^3,8^ |
|  | p.R899P | [rs137852752](http://www.ncbi.nlm.nih.gov/projects/SNP/snp_ref.cgi?rs=rs137852752) | Germline | ^3,10,11^ |
|  | p.A24E | [rs370120266](http://www.ncbi.nlm.nih.gov/projects/SNP/snp_ref.cgi?rs=rs370120266) | Germline | ^3,9^ |
|  | p.N903S | [rs373725296](http://www.ncbi.nlm.nih.gov/projects/SNP/snp_ref.cgi?rs=rs373725296) | Germline | ^3,8^ |
|  | p.E427D | Not found | Not reported | - |
| ***CAV1*** | p.V155I | [rs150368249](http://www.ncbi.nlm.nih.gov/projects/SNP/snp_ref.cgi?rs=rs150368249) | Not reported | - |
| ***KCNA5*** | p.E211D | [rs35853292](http://www.ncbi.nlm.nih.gov/projects/SNP/snp_ref.cgi?rs=rs35853292) | Unknown | ^12^ |
|  | p.G182R | rs755408841 | Germline | ^13^ |
| ***SMAD4*** | p.N13S | [rs281875323](http://www.ncbi.nlm.nih.gov/projects/SNP/snp_ref.cgi?rs=rs281875323) | Germline | ^3,14^ |
| ***SMAD9*** | p.K43E | [rs397514715](http://www.ncbi.nlm.nih.gov/projects/SNP/snp_ref.cgi?rs=rs397514715) | Germline | ^14^ |
|  | p.R294 | [rs397514716](http://www.ncbi.nlm.nih.gov/projects/SNP/snp_ref.cgi?rs=rs397514716) | Germline | ^14,15^ |
| ***TBX4*** | p.A35V | [rs148424252](http://www.ncbi.nlm.nih.gov/projects/SNP/snp_ref.cgi?rs=rs148424252) | Germline | ^16^ |
|  | p.Y382S | [rs536406541](http://www.ncbi.nlm.nih.gov/projects/SNP/snp_ref.cgi?rs=rs536406541) | Not reported | - |
| ***TOPBP1*** | p.S817L | [rs17301766](http://www.ncbi.nlm.nih.gov/projects/SNP/snp_ref.cgi?rs=rs17301766) | Not reported | - |
|  | p.R309C | [rs55633281](http://www.ncbi.nlm.nih.gov/projects/SNP/snp_ref.cgi?rs=rs55633281) | Not reported | - |
|  | p.N1042S | [rs10935070](http://www.ncbi.nlm.nih.gov/projects/SNP/snp_ref.cgi?rs=rs10935070) | Not reported | - |
| ***ENG*** | p.G214S | [rs150932144](http://www.ncbi.nlm.nih.gov/projects/SNP/snp_ref.cgi?rs=rs150932144) | Germline | ^3,17^ |
|  | p.G545S | rs142896669 | Germline | ^3,17^ |

References:

1. Austin ED, Loyd JE, Phillips JA. Heritable Pulmonary Arterial Hypertension. In: Adam MP, Ardinger HH, Pagon RA, et al., eds. *GeneReviews®*. Seattle (WA): University of Washington, Seattle; 1993. http://www.ncbi.nlm.nih.gov/books/NBK1485/. Accessed April 16, 2018.

2. Kabata H, Satoh T, Kataoka M, et al. Bone morphogenetic protein receptor type 2 mutations, clinical phenotypes and outcomes of Japanese patients with sporadic or familial pulmonary hypertension. *Respirol Carlton Vic*. 2013;18(7):1076-1082. doi:10.1111/resp.12117

3. Machado RD, Southgate L, Eichstaedt CA, et al. Pulmonary Arterial Hypertension: A Current Perspective on Established and Emerging Molecular Genetic Defects. *Hum Mutat*. 2015;36(12):1113-1127. doi:10.1002/humu.22904

4. Pfarr N, Szamalek-Hoegel J, Fischer C, et al. Hemodynamic and clinical onset in patients with hereditary pulmonary arterial hypertension and BMPR2 mutations. *Respir Res*. 2011;12:99. doi:10.1186/1465-9921-12-99

5. Sztrymf B, Coulet F, Girerd B, et al. Clinical outcomes of pulmonary arterial hypertension in carriers of BMPR2 mutation. *Am J Respir Crit Care Med*. 2008;177(12):1377-1383. doi:10.1164/rccm.200712-1807OC

6. Liu D, Liu Q-Q, Eyries M, et al. Molecular genetics and clinical features of Chinese idiopathic and heritable pulmonary arterial hypertension patients. *Eur Respir J*. 2012;39(3):597-603. doi:10.1183/09031936.00072911

7. Girerd B, Montani D, Eyries M, et al. Absence of influence of gender and BMPR2 mutation type on clinical phenotypes of pulmonary arterial hypertension. *Respir Res*. 2010;11:73. doi:10.1186/1465-9921-11-73

8. Machado RD, Aldred MA, James V, et al. Mutations of the TGF-beta type II receptor BMPR2 in pulmonary arterial hypertension. *Hum Mutat*. 2006;27(2):121-132. doi:10.1002/humu.20285

9. Machado RD, Eickelberg O, Elliott CG, et al. Genetics and genomics of pulmonary arterial hypertension. *J Am Coll Cardiol*. 2009;54(1 Suppl):S32-42. doi:10.1016/j.jacc.2009.04.015

10. Sankelo M, Flanagan JA, Machado R, et al. BMPR2 mutations have short lifetime expectancy in primary pulmonary hypertension. *Hum Mutat*. 2005;26(2):119-124. doi:10.1002/humu.20200

11. Machado RD, Rudarakanchana N, Atkinson C, et al. Functional interaction between BMPR-II and Tctex-1, a light chain of Dynein, is isoform-specific and disrupted by mutations underlying primary pulmonary hypertension. *Hum Mol Genet*. 2003;12(24):3277-3286. doi:10.1093/hmg/ddg365

12. Biesecker Lab/Human Development Section - Submitter - ClinVar. https://www.ncbi.nlm.nih.gov/clinvar/submitters/1160/. Accessed April 24, 2018.

13. Invitae - Submitter - ClinVar. https://www.ncbi.nlm.nih.gov/clinvar/submitters/500031/. Accessed April 24, 2018.

14. Nasim MT, Ogo T, Ahmed M, et al. Molecular genetic characterization of SMAD signaling molecules in pulmonary arterial hypertension. *Hum Mutat*. 2011;32(12):1385-1389. doi:10.1002/humu.21605

15. Drake KM, Zygmunt D, Mavrakis L, et al. Altered MicroRNA processing in heritable pulmonary arterial hypertension: an important role for Smad-8. *Am J Respir Crit Care Med*. 2011;184(12):1400-1408. doi:10.1164/rccm.201106-1130OC

16. Illumina Clinical Services Laboratory - Submitter - ClinVar. https://www.ncbi.nlm.nih.gov/clinvar/submitters/504895/. Accessed April 24, 2018.

17. Pfarr N, Fischer C, Ehlken N, et al. Hemodynamic and genetic analysis in children with idiopathic, heritable, and congenital heart disease associated pulmonary arterial hypertension. *Respir Res*. 2013;14:3. doi:10.1186/1465-9921-14-3
